# Coordination of the Uptake and Metabolism of Amino Acids in *Mycobacterium tuberculosis*-Infected Macrophages

**DOI:** 10.3389/fimmu.2021.711462

**Published:** 2021-07-13

**Authors:** Qingkui Jiang, Lanbo Shi

**Affiliations:** Public Health Research Institute, New Jersey Medical School, Rutgers Biomedical and Health Sciences, Rutgers The State University of New Jersey, Newark, NJ, United States

**Keywords:** amino acid transporters, arginine metabolism, glutaminolysis, immunometabolism, M1 polarization, *Mycobacterium tuberculosis*, redox homeostasis, system L transporters

## Abstract

Macrophage polarization to the M1-like phenotype, which is critical for the pro-inflammatory and antimicrobial responses of macrophages against intracellular pathogens, is associated with metabolic reprogramming to the Warburg effect and a high output of NO from increased expression of NOS2. However, there is limited understanding about the uptake and metabolism of other amino acids during M1 polarization. Based on functional analysis of a group of upregulated transporters and enzymes involved in the uptake and/or metabolism of amino acids in *Mycobacterium tuberculosis*-infected macrophages, plus studies of immune cell activation, we postulate a coherent scheme for amino acid uptake and metabolism during macrophage polarization to the M1-like phenotype. We describe potential mechanisms that the increased arginine metabolism by NOS2 is metabolically coupled with system L transporters LAT1 and LAT2 for the uptake of neutral amino acids, including those that drive mTORC1 signaling toward the M1-like phenotype. We also discuss the underappreciated pleiotropic roles of glutamine metabolism in the metabolic reprogramming of M1-like macrophages. Collectively, our analyses argue that a coordinated amino acid uptake and metabolism constitutes an integral component of the broad metabolic scheme required for macrophage polarization to M1-like phenotype against *M. tuberculosis* infection. This idea could stimulate future experimental efforts to elucidate the metabolic map of macrophage activation for the development of anti-tuberculosis therapies.

## Introduction

Macrophage activation, in response to infection by intracellular pathogens or to treatment with lipopolysaccharide (LPS) and/or interferon-γ (IFN-γ), results in M1-like or M1 phenotype characterized by high level-expression of pro-inflammatory and antimicrobial molecules (1–3). One of the key events accompanying M1 polarization is a programmed metabolic remodeling to the glycolysis pathway, reminiscent of the Warburg effect in cancer cells, to meet the increasing demand for energy, NADPH, and precursors for cell growth, differentiation and synthesis of effector molecules (4–6). This metabolic reprograming is an essential part of the defense mechanism of macrophages against infection by intracellular pathogens, such as *Mycobacterium tuberculosis* (*Mtb*) (7–9), the etiological agent of tuberculosis. Enhanced arginine uptake by the M1-like macrophage and its metabolism by the inducible nitric oxide synthase 2 (NOS2), which results in production of large amounts of NO, is indispensable for controlling many intracellular pathogens, including *Mtb* (10–12). Emerging evidence indicates that there is also an increased need for other amino acids, in particular those essential, for multiple cellular processes of activating immune cells, including energy metabolism, protein synthesis, redox balance, and cell growth and differentiation (13–16). For example, the uptake and metabolism of the essential neutral amino acids leucine and methionine are critical for clonal expansion and differentiation of effector T cells (17, 18). In addition, elevated tryptophan catabolism in macrophages due to *Mtb*-induced expression of indoleamine 2,3-dioxygenase 1 represses the ability of host immune cells to control the infection *via* inhibition of T cell proliferation (19). Despite this progress, our overall understanding of amino acid uptake mechanism and the contribution of their metabolism to the metabolic reprogramming required for macrophage polarization remains sketchy.

Membrane amino acid transporters mediate the uptake of amino acids from the extracellular milieu and/or their transfer between cellular compartments during immune cell activation (15). Abnormal expression and function of certain amino acid transporters are linked to a wide range of pathologies (20). Based on their sequence similarity or substrate specificity and transport mechanism, amino acid transporters are classified into various solute carrier (SLC) families or into various transport systems, respectively (20, 21). Among these, the SLC7 family comprises cationic amino acid transporters (CATs) of the y+ system and members of the L-type amino acid transporter (LAT) system, the y^+^L system, and the $x_{c}^{-}$ system (22). CATs mediate sodium-independent transport of cationic L-amino acids, such as arginine and lysine (23). Transporters of system L, y+L, and $x_{c}^{-}$ all function with a glycoprotein, 4F2HC, encoded by *SLC3A2/Slc3a2*, thereby forming the heteromeric amino acid transporters (HATs) (24, 25). HATs mediate exchange at a 1:1 stoichiometry with a broad spectrum of substrates that range from neutral amino acids [in the case of LAT1 (SLC7A5) and LAT2 (SLC7A8)] to negatively charged amino acids [in the case of the $x_{c}^{-}$ system xCT (SLC7A11)]. Given that HATs require intracellularly accumulated amino acids and/or metabolites as export substrates for the exchange of amino acids from the extracellular milieu, their upregulation during immune cell activation suggests a close functional association of HATs with the metabolic program of activating immune cells. For example, LAT1-mediated uptake of large, essential neutral amino acids, including leucine, is required for the metabolic reprogramming of effector T cells to support their proliferation and differentiation (18). Moreover, upregulated *xCT* expression helps maintain redox balance and prolong the survival of activated macrophages by mitigating damage caused by oxidative stress (26). However, the expression and function of amino acid transporters in amino acid uptake and metabolism during macrophage polarization are still understudied.

Based on the biochemical properties of amino acid transporters and emerging evidence from transcriptomics and metabolomics studies of activation of bone marrow-derived macrophages (BMDMs), such as by infection with *Mtb* or with LPS treatment (27–30), we propose the idea that increased expression of several types of transporters and enzymes involved in the uptake and/or metabolism of amino acids are metabolically coordinated to support macrophage polarization toward the M1-like phenotype. We discuss potential mechanisms that a functional coupling between increased arginine metabolism and system L transporters (LAT1 and LAT2) drives the uptake of neutral amino acids, including those required for M1 polarization. We further discuss the role of glutamine metabolism in the metabolic reprogramming of M1-like macrophages, which has usually been associated with M2 rather than with M1 macrophages.

## Arginine Uptake, Metabolism and Regeneration

As a conditionally essential amino acid, arginine plays an important role in innate and adaptive immunity. Its uptake from the extracellular milieu, mediated by cationic amino acid transporter 2 (CAT2), a member of system y+ (31), functions as a critical regulator of immunity under inflammatory conditions (32, 33). *Cat2* deficiency is associated with poor macrophage activation phenotypes, resulting in decreased NO production from NOS2 in M1 macrophages, as well as reduced production of polyamines and proline by arginase 1 in M2 macrophages (12, 34). Moreover, during *in vivo* infection with pathogens that induce highly polarized Th2 or Th1 responses (33), CAT2 is a critical factor for the development of protective Th1-dependent immunity (33, 35), suggesting an important role of extracellular arginine for expression of host adaptive immunity.

**Figure 1:**
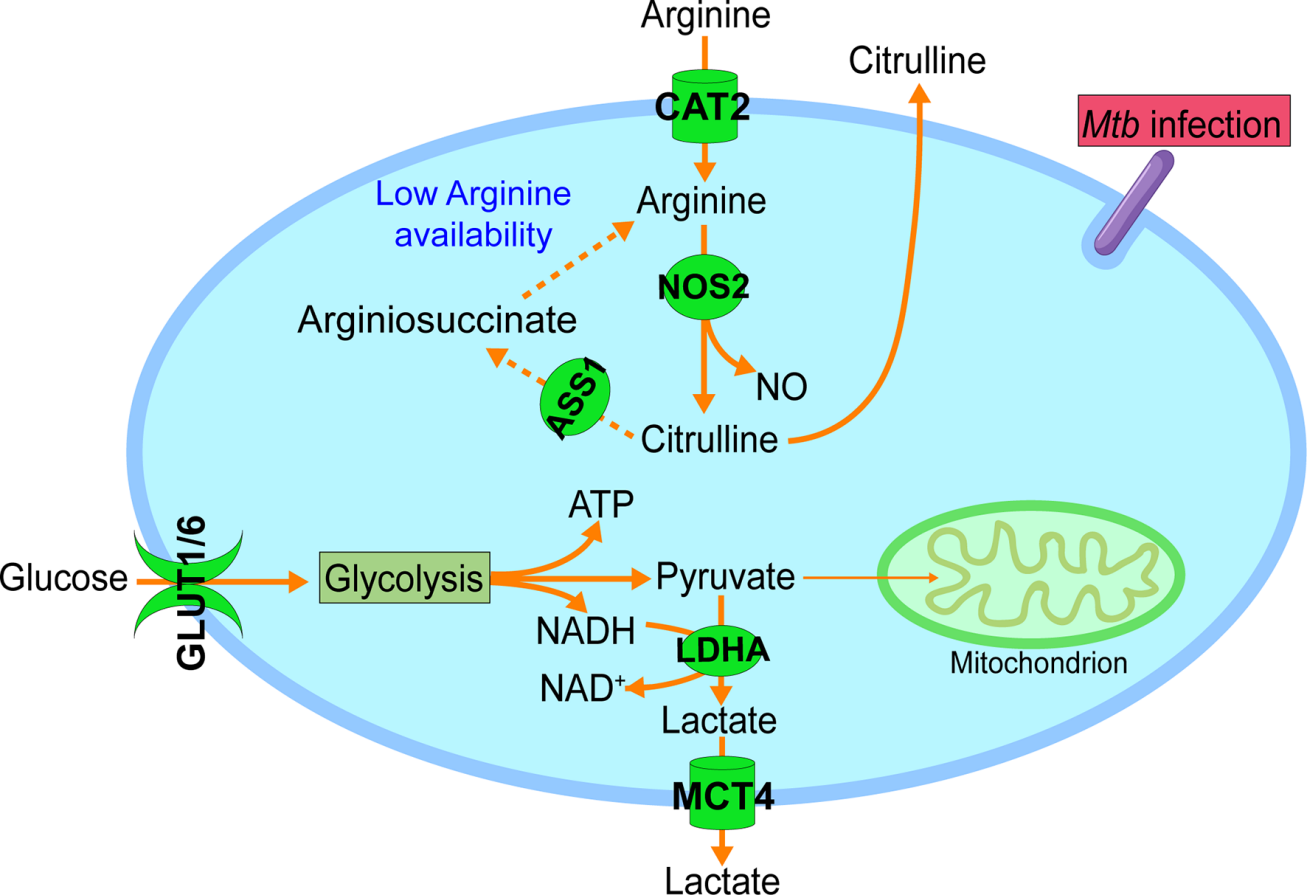
Increased arginine uptake and metabolism during M1-like polarization. In addition to the metabolic reprogramming to glycolysis with increased glucose uptake (GLUT1 & GLUT6) and lactate formation (LDHA) and secretion (MCT4), M1-like polarization is also accompanied by increased uptake and metabolism of arginine. Increased expression of cationic amino acid transporter 2 (CAT2) mediates the import of arginine from the extracellular microenvironment. Intracellular arginine is mainly catabolized by upregulated inducible nitric oxide synthetase 2 (NOS2) to NO and citrulline, and the latter is actively secreted to the extracellular microenvironment. With the decreasing availability of extracellular arginine, NOS2-derived citrulline is recycled for the regeneration of intracellular arginine *via* argininosuccinate synthetase 1 (ASS1) (dashed line), which will sustain the NO production by NOS2. GLUT1, glucose transporter 1; GLUT6, glucose transporter 6; LDHA, lactate dehydrogenase A; MCT4, monocarboxylate transporter 4 (MCT4). Data were derived from references and/or supplementary files therein (27–30).

Transcriptomics data from *Mtb-*infected BMDMs reveals a robust upregulation in the expression of *Cat2* within 4 - 12 hours of infection (27–29, 36). Since *Cat2* upregulation is concurrent with the timing of M1-like polarization and upregulation of *Nos2 (* 28, 29), as well as with metabolic reprogramming to the Warburg effect (7, 28, 36–38), CAT2 may have an important role in *Mtb* induced M1-like polarization (**Figure 1**). This notion is supported by NO production from NOS2 of M1 macrophages or IFN-γ–stimulated BMDMs infected with *M. bovis* BCG coming predominantly from arginine taken up by CAT2, and accompaniment of active secretion of most of citrulline, the product of NOS2, into the extracellular milieu (39, 40) (**Figure 1**). During mycobacterial infection *in vivo*, the drastic increase of citrulline in infected lungs, which is associated with the expression of host adaptive immunity (41), is also consistent with the essential role of NOS2 in the mediation of the antimycobacterial response.

The importance of arginine in immune cell function is further supported by the observation that activated immune cells actively recycle citrulline for the regeneration of intracellular arginine. Citrulline recycling involves the induction of argininosuccinate synthetase 1 (ASS1), which catalyzes the formation of argininosuccinate from citrulline and aspartate. Argininosuccinate is further lysed by argininosuccinate lyase (ASL) for the production of intracellular arginine (13, 39, 42). Under conditions of low arginine availability in the microenvironment, such as in infection foci, citrulline recycling is important for the antimycobacterial response by supporting effector T cell function and sustaining NO production from activated macrophages (41, 43). The upregulation of *Ass1* in *Mtb*-infected BMDMs (27, 29), probably indicates decreasing availability of extracellular arginine as a consequence of early *Cat2* induction, would thus warrant the supply of intracellular arginine for sustained NO production by NOS2 in activated macrophages (39) (**Figure 1**). This notion is supported by the observation that *Ass1*-deficient macrophages fail to salvage citrulline in arginine-scarce conditions, leading to their inability to control mycobacteria infection (39).

Increased LAT1 expression has been reported to be associated with citrulline transport to support the effector function of T cells (42). However, little is known about the metabolic and physiological roles of active transport of citrulline across the cell membrane in the context of macrophage polarization. Very interestingly, findings from a recent study using peritoneal macrophages have revealed additional roles of arginine metabolism in anti-*Mtb* macrophage function, which is related to mechanistic target of rapamycin (mTOR) activation and independent of NO production (44). In the following sections, we propose potential mechanisms by which uptake of essential neutral amino acids that are important for M1 polarization are metabolically coupled with the active production and secretion of citrulline to support macrophage polarization to the M1-like phenotype. We further discuss the involvement of glutamine metabolism in the metabolic reprogramming of M1-like macrophages.

## Uptake of Neutral Amino Acids by LAT1 and LAT2

**Figure 2:**
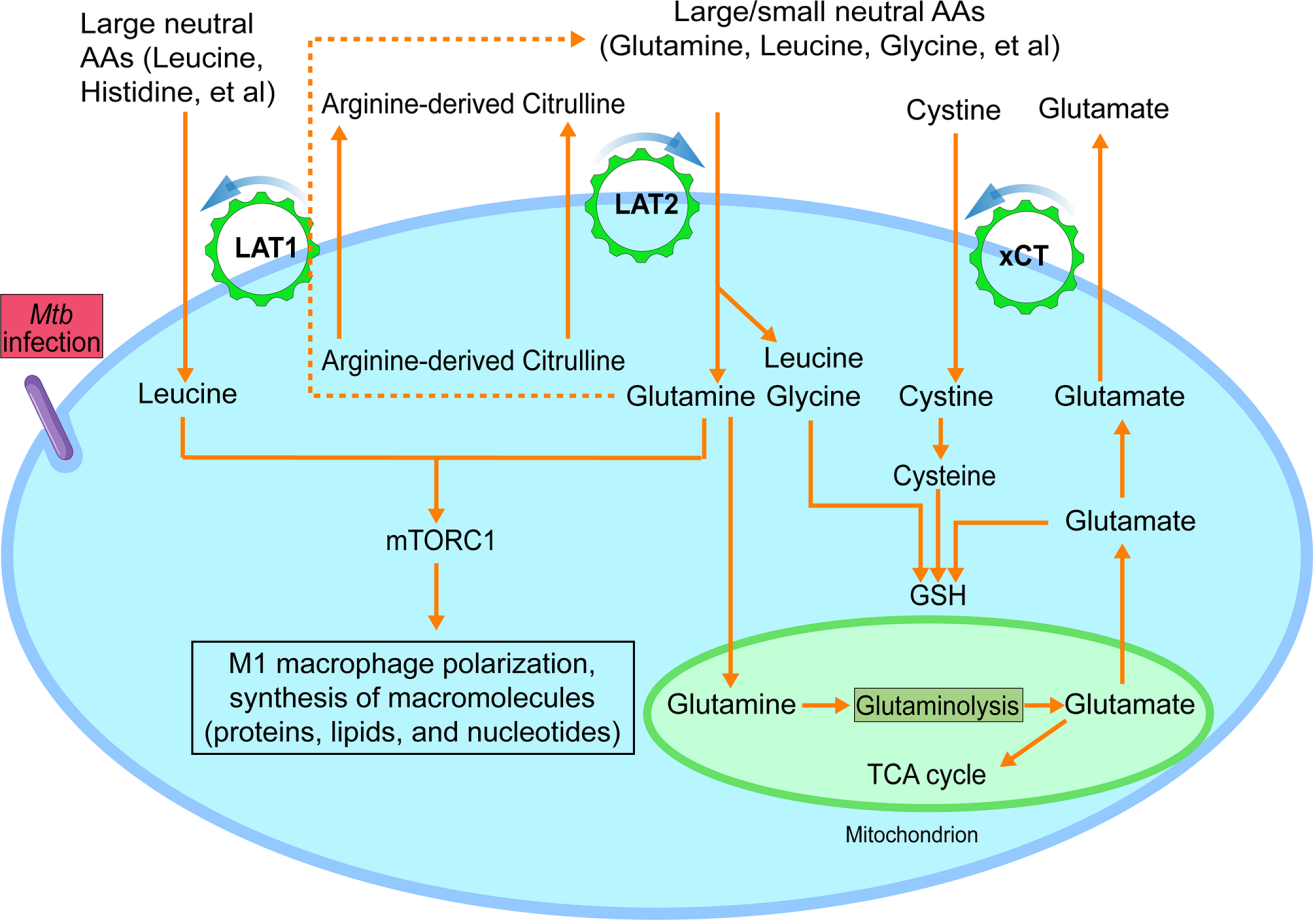
Functional model of LAT1, LAT2 and xCT amino acid transporters during M1-like polarization. During M1-like polarization, increased need for amino acids, including essential ones, is met by upregulated amino acid transporters LAT1, LAT2 and xCT. LAT1 and LAT2-mediated uptake of neutral amino acids from the extracellular environment requires an intracellularly accumulated neutral amino acid as export substrate to activate the exchange process with a 1:1 stoichiometric ratio. And such intracellular metabolite could be citrulline, which is abundantly produced by the highly upregulated NOS2 and actively secreted by M1-like macrophages. The uptake of neutral amino acids, such as leucine and glutamine, drives the activation of mTORC1, which is critical for multiple processes of M1-like polarization, including synthesis of macromolecules. Alternatively, the apparent differential affinities of LAT1 and LAT2 with certain neutral amino acid substrates also suggest a potential functional cooperation between the two LAT transporters in which LAT2-mediated accumulation of intracellular glutamine could serve as an export substrate of LAT1 (dashed line), as seen in cancer cells, to drive the uptake of essential neutral amino acids for which LAT1 has high affinity, such as histidine. The uptake of cystine by xCT, activated by increased production of glutamate from mitochondrial glutaminolysis with increased GLS (glutaminase) and the reduction of intracellular cystine to cysteine, participates in glutathione (GSH) synthesis, contributing to the redox homeostasis of M1-like macrophages. Findings from recent report that arginine metabolism-mediated glycolysis and mTOR activation are required for *Mtb* control in the absence of NO support the additional role of arginine metabolism, as reported in (44). AA, amino acids. Data were derived from references and/or supplementary files therein (27–30).

In addition to arginine, immune cell activation requires essential neutral amino acids for effector functions. Uptake of these amino acids is usually associated with increased expression of particular system L and/or y+L transporters. The system L transporters LAT1 and LAT2 mediate obligatory Na^+^-independent neutral amino acid exchange with a 1:1 molar ratio (**Figure 2**). They have overlapping (but not identical) substrate selectivity, and their affinities for a given amino acid on the intracellular side of the cell membrane are much lower than those on the extracellular side (25, 45), thus favoring influx of their substrates. LAT1 has an apparent specificity for large neutral amino acids, including essential large, branched neutral amino acids, such as leucine and isoleucine (46). In contrast, LAT2 has broad substrate selectivity for both large and small neutral amino acids (47–49). In addition, these two LAT transporters have quite different apparent affinities for several amino acid substrates. For example, in contrast to LAT1, LAT2 has an apparent high affinity for glutamine and a low affinity of histidine (50). These findings underscore the potentially distinct roles of these two system L transporters for the uptake of particular neutral amino acids (**Figure 2**).

In *Mtb*-infected BMDMs, concurrent with the upregulation of *Cat2*, the early induction of *Slc7a5*, *Slc7a8* and *Slc3a2* (27–29) suggests increased uptake of neutral amino acids by both LAT1/4F2hc and LAT2/4F2hc during M1-like polarization (**Figure 2**). LAT1-mediated uptake of the essential neutral amino acid leucine and its signaling are indispensable for activation of serine-threonine kinase complex mTORC1 and the subsequent metabolic reprogramming, clonal expansion, and effector differentiation of activated T cells (18). Similarly, the LAT1-mediated uptake of methionine, another essential neutral amino acid, functions as a main rate-limiting step during T cell activation for protein synthesis and the methionine cycle, which supplies methyl donors for the dynamic nucleotide methylation and epigenetic reprogram (17, 51). Still unknown are the specific amino acid substrates of LAT2 and their distinct functions in the metabolic reprogramming of immune cells. However, the apparent high affinity of LAT2 for certain amino acids, such as glutamine (50) plus its upregulation in *Mtb*-infected BMDMs, suggest a potential role for LAT2 in the uptake of distinct neutral amino acids that are important for M1-like polarization. Thus, increased expression of both LAT1 and LAT2 during *Mtb* infection of macrophages would meet the demand of M1-like macrophages for various neutral amino acids, especially essential ones.

An open question regarding the operational mechanism of system L transporters is the identity of intracellularly accumulated neutral amino acids that are needed to activate the system L transporters by serving as their efflux substrate (45). Since LAT1-mediated citrulline transport is critical for the activation of effector CD4^+^ and CD8^+^ cells (42), it is plausible that citrulline production and its active secretion across the cell membrane of *Mtb* induced M1-like macrophages fulfill similar important metabolic functions during macrophage activation, as discussed in the following sections.

## xCT for Redox Homeostasis

Activation of innate and adaptive immune cells to the pro-inflammatory phenotype is associated with production of reactive oxygen and nitrogen species, molecules that are important for redox signaling and regulation of immune responses (52). Maintaining redox balance of activated immune cells is essential for preventing the deleterious effects of cytotoxic bioactive molecules. Synthesis of glutathione (GSH), a key small-molecule intracellular antioxidant, functions as one of the critical modulators of intracellular redox homeostasis by countering the increased levels of oxidative stress of activated immune cells (26, 53, 54). GSH synthesis is associated with increased expression of xCT, which, together with 4F2hc, mediates Na^+^-independent and electroneutral import of extracellular anionic cystine with high affinity against export of intracellular glutamate (55) (**Figure 2**). The exchange is driven by intracellular reduction of cystine to cysteine and the high concentration of intracellular glutamate (56, 57). Cysteine, the reduced form of cystine, together with glutamate and glycine, participates in the synthesis of GSH (**Figure 2**).

*Mtb* infection of BMDMs leads to an increasing demand for GSH to protect activated macrophages from the cytotoxic bioactive molecules. Consequently, expression of genes encoding xCT for cystine import and enzymes involved in cystine reduction and glutathione synthesis is increased to meet this demand (36). As for the other substrates for GSH synthesis, glycine, a small neutral amino acid, can be imported by LAT2 (49), and it can also be produced from glycolysis intermediate 3-phosphoglycerate-derived intracellular serine (58). Glutamate can come predominantly from the glutaminolysis pathway in mitochondria (**Figure 2**). This is consistent with the upregulation of *Gls/GSL* in *Mtb*-infected BMDMs or human macrophages (27, 28, 59), whose product glutaminase catalyzes a key rate-limiting step of glutaminolysis with the formation of glutamate. We also observed a rapid and extensive depletion of glutamine from the culture medium during the early phase of BMDM infection by *Mtb* (up to 12 hours post-infection, corresponding to the M1-like polarization) in comparison to a small amount of glutamate secreted into the culture medium (our unpublished data). The requirement of glutamine for the macrophage response to *Mtb* infection is further demonstrated by findings that human monocyte-derived macrophages (hMDMs) display decreased cytokine responses when cultured in medium devoid of glutamine or with the inhibition of glutamine utilization by glutaminase-specific inhibitors, such as BPTES and C968 (59).

As the most abundant and versatile amino acid in the body, glutamine is consumed by immune cells at a rate similar to or higher than glucose. Glutamine has been reported to be mainly associated with M2 macrophages based on transcriptional and metabolic profiling and analysis (60). However, the sampling and analysis that were carried out at or after 24 hr-treatment of BMDMs by LPS and IFN-γ (for M1) or IL-4 (for M2) could have skewed the conclusion of glutamine metabolism toward an association with M2 polarization. This is because, as shown by transcriptional (28, 36) and metabolomics studies [supplemental data file of dynamic changes of metabolites during macrophage polarization in (30)], macrophage polarization to the M1-like phenotype, either induced by pathogen infection or by ligand signaling, such as by LPS and/or IFN-γ, occurs at up to 12 hrs post-treatment or infection. Thus, analyses carried out at or beyond 24 hrs post-treatment may not fully represent the immunometabolic state of M1-like but rather a transitioning state toward M2 macrophages, as evidenced by the expression of M2 markers (60). The different signaling pathways involving *Mtb* or LPS that resulted in differential immunometabolic properties of hMDMs, as reported (37), could be a contributing factor for the differential response in these cells. Involvement of glutamine in M1-like polarization is shown by increased enrichment of the tricarboxylic acid (TCA) cycle intermediate ^13^C4 succinate from ^13^C5 glutamine metabolomics study in LPS-activated BMDMs at 3 hrs post treatment (4); and glutamine-derived succinate functions as signaling molecules to promote the HIF-1α-mediated metabolic reprogramming of macrophages to glycolysis and proinflammation (4, 61). In proliferating cells, glutamine fulfills pleiotropic functions, including anaplerotic TCA cycle substrates, nucleotide synthesis, and synthesis of aspartate, to support cell growth and cellular redox homeostasis (62, 63). The observed increased dependence on glutamine in *Mtb*-infected hMDMs or THP1 cells at 18 hrs p.i supports the involvement of glutamine in macrophage activation, while the contribution of glutamine to macrophage polarization to the M1, M2 or both was not clearly defined (64). The specific pathways involving glutamine metabolism contributing to the M1 and/or M2 polarization can be further dissected in future studies using therapeutic compounds, such as small molecule inhibitors BPTES, C968 and CB-839 targeting glutaminase (59, 62), and ^13^C and ^15^N glutamine isotope tracing metabolomics.

## Metabolic Interplay Between Arginine Metabolism and System L Transporters During M1-Like Polarization

Uptake of amino acids by various membrane transporters has been postulated to be a coordinated process in response to the metabolic and physiological demands of the cells. It is well known that apart from glucose, essential neutral amino acids are indispensable for cell growth and duplication during cell proliferation (65). Their uptake from the extracellular milieu is usually associated with increased expression of membrane transporters, including system L transporters and a neutral amino acid transporter SLC1A5 (66, 67). Functional coupling between LAT1 and SLC1A5 has been identified as a critical process for the unidirectional uptake of essential neutral amino acids required for cell growth and proliferation in cancer cells. Specifically, SLC1A5 mediates the influx of glutamine, which then activates the uptake of large essential amino acids, including leucine, by LAT1 by serving as its intracellular efflux substrate (67, 68). Consequently, the influx of large essential amino acids, such as leucine, together with glutamine, drives the mTORC1 activation and the metabolic reprogramming required for cell growth (68–70).

During *Mtb* infection, the downregulation of *Slc1a5/SLC1A5* in both murine and human (27, 59) suggests that SLC1A5 may not be the major transporter for the uptake of glutamine in M1 macrophages. Instead, the increased expression of *Slc7a8/Lat2* at early stage of macrophage infection from our analysis, the high affinity of LAT2 for glutamine (50), and the critical role of LAT2 in glutamine-dependent mTOR activation for the metabolic programming of cancer cells (71), suggest that LAT2 could be a main player in glutamine uptake by *Mtb* induced M1-like macrophages. Since LAT2 function requires an intracellularly accumulated neutral amino acid as an efflux substrate for the uptake of glutamine and other neutral amino acids, an ideal and metabolically suitable candidate that meets the criteria could be citrulline. This compound is made in abundant quantity by upregulated NOS2 and actively secreted to the extracellular milieu by M1 or M1-like macrophages (39, 40) [also refer to supplemental data in (30)]. The uptake of glutamine *via* LAT2-mediated citrulline/glutamine exchange could also activate LAT1 for the uptake of other large essential amino acids, especially those for which LAT1 has high affinity, by serving as its efflux substrate (**Figure 2**). This model of amino acid uptake *via* antiporters, such as system L transporters involving abundant glutamine for the uptake of essential amino acids, is consistent with the notion that glutamine serves as the cellular currency for the exchange (72). Another probable functional coupling scenario between arginine metabolism and system L transporters is that citrulline can also serve directly as the export substrate of LAT1 for the uptake of essential amino acids, given that citrulline has been identified as a substrate of LAT1 (42) (**Figure 2**). Thus, the working model for the functional coordination between arginine metabolism and system L transporters specifies that the activation of both LAT1 and LAT2 transporters by NOS2-derived citrulline, as an exchange substrate, results in the uptake of neutral amino acids, especially essential ones. This in turn leads to the activation of the mTORC1 pathway, such as by leucine and glutamine (68, 69) and metabolic reprogramming of M1-like macrophages (**Figure 2**). Indeed, the observations that arginine metabolism-mediated glycolysis and mTOR activation are required for *Mtb* control in the absence of NO support the additional role of arginine metabolism (44), as proposed in our model (**Figure 2**). Future studies combining genetics, metabolomics, and pharmacological approaches will be necessary to validate the proposed functional coupling of LAT transporters and arginine metabolism during M1 polarization. Since activated macrophages vigorously recycle citrulline when extracellular arginine level is low (43), it also will be interesting to investigate the specific role of these transporters in citrulline import in order to generate intracellular arginine.

## Concluding Remarks

Our analyses suggest that functional cooperation among various amino acid transporters in the uptake and metabolism of amino acids constitutes an integral component of the metabolic remodel program of M1-like macrophages. In particular, the involvement of glutamine in the metabolic reprogramming during macrophage activation underscores the important role of glutamine as an important carbon and nitrogen source in M1-like macrophages. This immunometabolic switch accompanying M1-like polarization at early phase of host-pathogen interaction is in agreement with reduction of *Mtb* CFU in infected BMDMs (73). Thus, our understanding offers novel mechanistic insights into the broad metabolic remodeling program required for macrophage-mediated immunity, in addition to the known importance of the Warburg effect. However, our analysis doesn’t address the mechanism by which *Mtb* modulates the immunometabolic switch required for the pro-inflammatory and antimicrobial response of macrophages to survive and/or persist in host cells, as shown in a recent study (74), given the limited studies in this rapidly evolving area of research. Further, given that different types of macrophages, in particular recruited *vs.* resident macrophages, are known to show variable degrees of immunometabolic responses to *Mtb* infection and/or stimuli (8, 74–78), and that macrophage response to infection and/or stimuli show a dynamic defending and resolution/adaptation process (28, 36, 64, 79, 80), cautions are warranted to interpret the immunometabolic programs of different macrophages at different stages of the infection and/or in responding to different stimuli. A similar metabolic coupling scenario in the uptake and metabolism of amino acids likely exists in *in vivo* settings, given that a similar set of genes, including *Cat2, Nos2, Ass1, Lat1, Lat2*, *xCT* and *Gls* was also upregulated in *Mtb*-infected mouse lungs, along with the onset of host adaptive immunity (our unpublished observations). Such notion can be dissected in future studies in the context of the complex microenvironment of *Mtb*-infected lungs. In particular, the necessity for glucose and other nutrients, including amino acids, required for the expression of antimicrobial and pro-inflammatory response likely varies among different immune cell types/subtypes in the microenvironment at different stages of the infection, which may manifest a fine-tuning of coordination and/or competition among nitrogen and carbon metabolic pathways of activated immune cells. These considerations should be taken into account to define the immunometabolic features of different immune cells in the *in vivo* settings. A better understanding of metabolic strategies of immune cell activation will lead to the development of immunometabolic therapeutics with enhanced efficacy against *Mtb* infection.

## Data Availability Statement

The gene expression data were derived from publicly available datasets deposited in the Genome Expression Omnibus (https://www.ncbi.nlm.nih.gov/geo/) under accession numbers GSE31734 and GSE79733, and the supplementary data in (29).

## Author Contributions

LS conceived the concept and designed the manuscript outline. LS and QJ drafted the manuscript and prepared the figures. All authors contributed to the article and approved the submitted version.

## Funding

The work was supported by National Institutes of Health (NIH) grant R01AI127844.

## Conflict of Interest

The authors declare that the research was conducted in the absence of any commercial or financial relationships that could be construed as a potential conflict of interest.
